# Frugal innovation in the business environment: a literature review and future perspectives

**DOI:** 10.3389/frai.2024.1385522

**Published:** 2024-12-06

**Authors:** Carlos Escudero-Cipriani, Julio García-del Junco, Raquel Chafloque-Céspedes, Aldo Alvarez-Risco

**Affiliations:** ^1^Doctoral Program in Strategic Management and International Business, Universidad de Sevilla, Seville, Spain; ^2^Department of Business Administration and Marketing, Universidad de Sevilla, Seville, Spain; ^3^Escuela de Posgrado, Universidad San Ignacio de Loyola, Lima, Peru; ^4^Facultad de Administración y Negocios, Universidad Tecnológica del Perú, Lima, Peru

**Keywords:** frugal innovation, product development, management practices, artificial intelligence, business environment, systematic review

## Abstract

**Introduction:**

This research aims to explore the growing field of frugal innovation within the business environment, particularly its intersection with sustainability and artificial intelligence.

**Methods:**

Through a comprehensive literature review, the study analyzes key research trends and methodologies from 420 scholarly articles published between 2012 and August 2024. A bibliometric review traces the evolution of frugal innovation, while a content analysis provides insights into its practical applications across various industries, especially in resource-constrained settings.

**Results:**

The findings highlight the significant role of frugal innovation in addressing global challenges, such as reducing environmental impact and promoting social inclusion, especially through the adoption of cleaner technologies and socially responsible business practices. The study also emphasizes the transformative potential of AI in enhancing the scalability and efficiency of frugal solutions.

**Discussion:**

This research contributes to the ongoing conversation on sustainable development by identifying knowledge gaps and proposing future strategies for leveraging frugal innovation to drive inclusive growth. The implications of this research are valuable for academics, practitioners, and policymakers aiming to foster sustainable innovation in diverse socio-economic contexts.

## Introduction

Frugal innovation, which is also known as Jugaad innovation, means, in the words of Hossain et al. (2016) “a resource-scarce solution (i.e., product, service, process, or business model) that is designed and implemented despite financial, technological, material or other resource constraints, whereby the outcome is significantly cheaper than competitive offerings (if available) and is good enough to meet the basic needs of customers who would otherwise remain un(der)serve.” Also, frugal innovation has become increasingly relevant in recent years. It involves finding creative, efficient, and affordable solutions to complex problems, most notably in contexts where resources are limited.

Likewise, Jugaad innovation is related to innovation in India. Thus, Jugaad is a Hindi term that, according to Radjou et al. (2012), means innovative improvement based on creativity and skills. Jugaad embodies the spirit of improvisation and innovative problem-solving, where people find ingenious solutions to everyday challenges by thinking outside the box and making the best use of available resources. Jugaad innovation means developing options, improvisations, and make-dos to overcome limited resources and fix seemingly insoluble problems (Prahalad and Mashelkar, 2010).

Recent research shows the need for more research on frugal innovation, as reported by Saleem et al. (2024), who focus on the relationship between frugal innovation and artificial intelligence and the Internet of Things (Qin, 2024). New research is also focused on evaluating the contribution of frugal innovation to digitalization and Sustainable Development (van Tuijl et al., 2024). In terms of the use of AI, Alfeir (2024) described the impact on relatives’ communication which can be used also by frugal innovators for their communication and even to be trained (Klarin et al., 2024).

Frugal innovation has three main components (Weyrauch and Herstatt, 2016): substantial cost reduction, focus on core functionalities, and optimized performance level. These pillars help to understand how this strategy makes it possible to offer affordable products and services without sacrificing quality and tailored to emerging or developed markets. a. Substantial cost reduction: Frugal innovation implies a significant decrease in production and procurement costs. This is achieved through the efficient use of resources and the elimination of unnecessary cost-increasing elements. For example, rather than focusing on developing advanced or luxurious technologies, frugal innovators create products that meet basic needs at a considerably lower price than existing alternatives on the market. This approach seeks to maintain affordability from the end customer’s perspective. b. Concentration on essential functionalities: Frugal innovation focuses on providing only the basic functions necessary to fulfill the product’s main purpose. By eliminating non-essential or ‘luxury’ features, the complexity and cost of manufacturing can be reduced without compromising the product’s functionality. This simplifies the production process and makes products more accessible and easier to use. The key is identifying which functions are indispensable to users and leaving out those that do not add essential value. c. Optimized level of performance: Unlike innovations that seek to maximize performance in all dimensions, frugal innovation optimizes performance to meet the specific requirements of the context in which the product will be used. This implies providing an adequate quality level to meet the customer’s expectations without over-engineering. The product must function robustly within the local conditions and specific needs without adding unnecessary additional costs. In this way, Table 1 shows the theoretical approach and their relation to ease of use, new product development, firm strategy, Sustainable Development Goals (SDG), and the industrial sector.

**Table 1:** Innovation theoretical approach and their relation to ease of use, new product development, firm strategy, Sustainable Development Goals (SDG), and the industrial sector.

Theoretical approach	Relation to ease of use	Relation to New Product Development (NPD)	Firm strategy	Relation to SDG	Industrial sector
Disruptive Innovation	Often leads to simpler, more accessible technologies	Drives NPD by encouraging simpler, low-end products for untapped markets	Forces companies to adapt strategies to disruptive market entrants	Helps address SDG 9 (Industry, Innovation, Infrastructure)	Technology, Consumer Electronics
Open Innovation	Improves ease by encouraging external ideas that refine usability	NPD relies on external partnerships and collaborations	Strategic shift toward collaboration, reducing R&D costs	SDG 17 (Partnerships for Goals)	Biotechnology, Pharmaceuticals, IT
Blue Ocean Strategy	Simplifies customer experience by creating uncontested markets	NPD focuses on offering unique, value-added products	Avoids competition through differentiation and creating new demand	Aligns with SDG 8 (Decent Work and Economic Growth)	Manufacturing, Automotive, Services
Diffusion of Innovation	Ease of use is critical for adoption, particularly among early adopters	Guides NPD in understanding user segments and adoption rates	Strategy focuses on the stages of adoption and tailoring product releases	Contributes to SDG 10 (Reduced Inequalities)	Technology, Consumer Goods, Agriculture
Radical Innovation	Usually more complex initially but can lead to breakthrough usability	NPD is driven by completely new technologies and concepts	High-risk, high-reward strategic approach to market leadership	Supports SDG 9 and SDG 7 (Affordable Clean Energy)	Renewable Energy, Space Exploration, Biotechnology
Incremental Innovation	Improves ease of use gradually through small updates	NPD focuses on continuous improvement rather than large leaps	Enhances existing strategies by improving efficiency	Contributes to SDG 12 (Responsible Consumption & Production)	Consumer Electronics, Automotive, Healthcare
User-Centered Innovation	Focuses on user feedback to enhance ease of use	NPD is directly shaped by user needs and feedback	Strategy involves continuous feedback loops to refine products	Supports SDG 3 (Good Health and Well-being)	Healthcare, Software, Consumer Goods
Technology Push Innovation	Ease of use may be secondary to technical advancement	NPD is driven by technological breakthroughs rather than user demand	Strategy focuses on R&D and pushing new technologies into the market	Aligns with SDG 9 and SDG 11 (Sustainable Cities)	IT, Advanced Manufacturing, Telecommunications
Market Pull Innovation	Ease of use is a priority to meet specific market demands	NPD is guided by consumer needs and market research	Strategy centers on satisfying existing market needs through innovation	Supports SDG 2 (Zero Hunger)	Food & Beverage, Consumer Electronics, Retail
Design Thinking	Emphasizes simplicity and user-centric designs	NPD focuses on prototyping and iterative development	Strategy involves iterative cycles to refine products and reduce risks	Contributes to SDG 4 (Quality Education) and SDG 9	Education, Healthcare, Technology

The convergence between frugal innovation and artificial intelligence is particularly important in the context of the United Nations’ Sustainable Development Goals (SDGs). SDG 9, which seeks to build resilient infrastructure, promote inclusive industrialization, and foster innovation, directly benefits from this synergy. Applying AI-powered frugal solutions can significantly contribute to closing infrastructure gaps, facilitating access to basic services, and promoting economic development in disadvantaged communities. Moreover, this combination of innovation and technology can also be instrumental in addressing other SDGs, such as eradicating poverty (SDG 1), improving health and well-being (SDG 3), and promoting quality education (SDG 4), among others. By enhancing communities’ capacity to solve their problems effectively and sustainably, frugal innovation powered by AI is positioned as a fundamental instrument in the search for more equitable and sustainable global development. Table 2 shows the relationship between Sustainable Development Goals (SDGs) 9, 1, 4, and 3 with frugal or Jugaad innovation and artificial intelligence (AI), emphasizing the relevant sub-themes for each SDG.

**Table 2:** Relationship between Sustainable Development Goals (SDGs) 1, 3, 4 and 9 with frugal or Jugaad innovation and artificial intelligence (AI), emphasizing the relevant sub-themes for each SDG.

SDG	Sub-themes	Connection to frugal (Jugaad) innovation	Connection to AI
SDG 1: No Poverty	Access to basic services Sustainable livelihoods Social protection	Promotes cost-effective products and services that cater to marginalized populations. Innovations such as affordable healthcare devices, clean energy solutions, and micro-entrepreneurship tools uplift communities out of poverty.	Identifies vulnerable populations through data analysis and offers personalized solutions for poverty alleviation programs. AI-powered platforms can also connect impoverished areas with employment and education opportunities.
SDG 3: Good Health and Well-being	Universal health coverage Access to essential medicines Disease prevention and health promotion	Develops low-cost healthcare devices, diagnostics, and telemedicine solutions that increase access to healthcare in rural and under-resourced regions, especially where infrastructure is lacking	Enhances healthcare delivery through predictive diagnostics, personalized medicine, and real-time health monitoring. AI-driven telemedicine platforms extend healthcare access to remote areas.
SDG 4: Quality Education	Inclusive education Access to quality learning resources Skills for future work	Creates low-cost, adaptable educational tools like mobile-based learning applications, ensuring access to education even in resource-constrained areas. This also supports skills development tailored to local needs.	Offers personalized learning experiences, language translation, and virtual tutors. AI also assists in automating administrative tasks, allowing teachers to focus on quality education.
SDG 9: Industry, Innovation, and Infrastructure	Sustainable industrialization Innovation Resilient infrastructure	Focuses on creating low-cost, efficient, and sustainable solutions, leveraging local resources and minimal infrastructure, ideal for developing economies where large-scale industrial investments are limited.	Optimizes processes for innovation, improving efficiency and scalability in industries. AI-driven predictive models aid in infrastructure maintenance and development.

This study significantly contributes to the academic literature on frugal innovation by advancing its theoretical understanding. First, it delves deeper into the key concepts of frugal innovation, allowing researchers and practitioners better to understand its nature and application in diverse contexts. Furthermore, the study presents an integrated map of the existing literature on the topic, using thematic and keyword analysis. This allows for a clearer view of the current state of knowledge and integrates contributions from different disciplines, enriching the understanding of frugal innovation by placing it within the broader context of innovation and management. In doing so, the article also identifies the most relevant theoretical and methodological foundations underpinning this area of research, providing future researchers with a solid reference to continue exploring the topic. Finally, by highlighting existing gaps in research, the study proposes a clear agenda for future work, highlighting areas that need further exploration and development to advance the field.

Innovation is the engine that drives human progress, a dynamic force that drives the evolution of society, the economy, and technology. Since the dawn of civilization, humanity has constantly sought ways to improve and optimize its tools, processes, and systems to meet changing needs and new challenges. In this modern era, marked by globalization and the technological revolution, innovation has become fundamental for survival and success in an increasingly competitive and complex world. The concept of innovation covers various activities and approaches, from creating revolutionary products to optimizing existing processes. However, beyond definitions and categorizations, the true essence of innovation lies in the ability to think creatively, challenge the status quo, and constantly seek new solutions to old problems. In this sense, innovation is not limited to technological companies or institutions but extends to all aspects of human life, from education and health to agriculture and transportation.

One of the most fascinating concepts to emerge in the field of innovation in recent decades is that of frugal innovation. This approach, originating in developing countries with limited resources, is based on doing more with less and finding ingenious and economical solutions to complex problems. Although frugal innovation is often associated with contexts of scarcity, its relevance and applicability extend far beyond the confines of developing economies. At its core, frugal innovation shares many fundamental principles of conventional innovation, such as creativity, adaptability, and a focus on user needs. However, what sets it apart is its emphasis on simplicity, efficiency, and accessibility. While traditional innovation is often associated with large investments in research and development, frugal innovation focuses on finding practical, cost-effective solutions that can be implemented quickly with limited resources. Frugal innovation has emerged as a distinctive business strategy today, challenging the conventional notion that innovation requires significant investments (Zeschky et al., 2011); instead of focusing on complexity and high costs, frugal innovation focuses on simplicity, efficiency, and resource optimization (Cai et al., 2019). Companies worldwide are using this approach to develop innovative solutions accessible and affordable to a broader audience (Khan and Melkas, 2020).

Table 3 shows the relationship between technology theoretical approach and their relation to innovation, Sustainable Development Goals (SDG), and the industrial sector.

**Table 3:** Technology theoretical approach and their relation to innovation, Sustainable Development Goals (SDG), and the industrial sector.

SDG	Relation to innovation	Relation to SDG	Relation to industrial sector
Technology Acceptance Model (TAM)	Drives innovation by emphasizing user-centric design to enhance technology adoption.	Supports SDG 9 (Industry, Innovation, and Infrastructure) by promoting tech use in sustainable industry development.	Relevant in sectors like manufacturing, IT, and healthcare, where ease of technology integration impacts productivity and adoption.
Unified Theory of Acceptance and Use of Technology (UTAUT)	Encourages innovation by aligning technology features with user expectations and social factors.	Enhances SDG 4 (Quality Education) and SDG 8 (Decent Work and Economic Growth) through widespread technology adoption.	Applicable in education, government, and corporate environments where tech adoption can improve service delivery and productivity.
Diffusion of Innovations Theory (DOI)	Focuses on the process of spreading innovations, crucial for early adopters and laggards	Supports SDG 11 (Sustainable Cities and Communities) by promoting the diffusion of eco-friendly innovations.	Used in sectors like energy, agriculture, and telecom, where the adoption of new technologies needs to be widespread for societal impact.
Task-Technology Fit (TTF) Theory	Directs innovation towards creating technologies that enhance task efficiency.	Linked to SDG 12 (Responsible Consumption and Production) by ensuring that tech supports sustainable production.	Key in industries such as logistics, healthcare, and financial services where task-specific technology drives efficiency and effectiveness.
Social Construction of Technology (SCOT)	Encourages innovation by involving diverse social groups in the development process.	Aligns with SDG 10 (Reduced Inequality) by ensuring technology meets diverse societal needs and reduces disparities.	Found in public services, social platforms, and communications, where societal impact and feedback shape technology adaptation and innovation.

**Figure 1:**
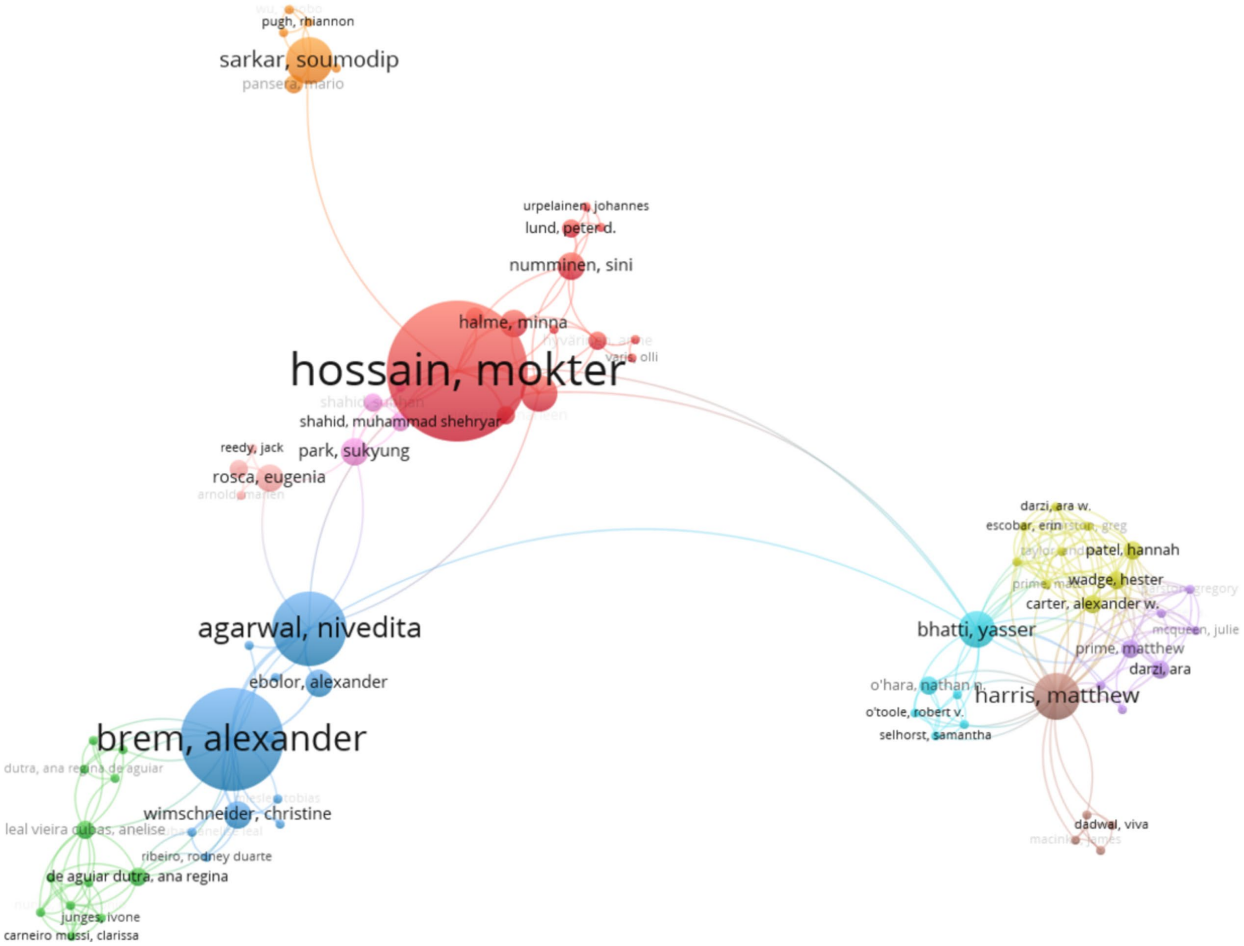
Cooperation network between authors during 2012–August 2024.

Figure 1 shows the map of collaboration among authors. It can be recognized that Mokter Hossain from Qatar University, Qatar, led the first cluster (red) (15 publications about the current topic evaluated). Alexander Brem led the second cluster (blue) from Universität Stuttgart, Germany (11 publications). Nivedita Agarwal led the third cluster (sky blue) from Universität Stuttgart, Germany (8 publications).

**Figure 2:**
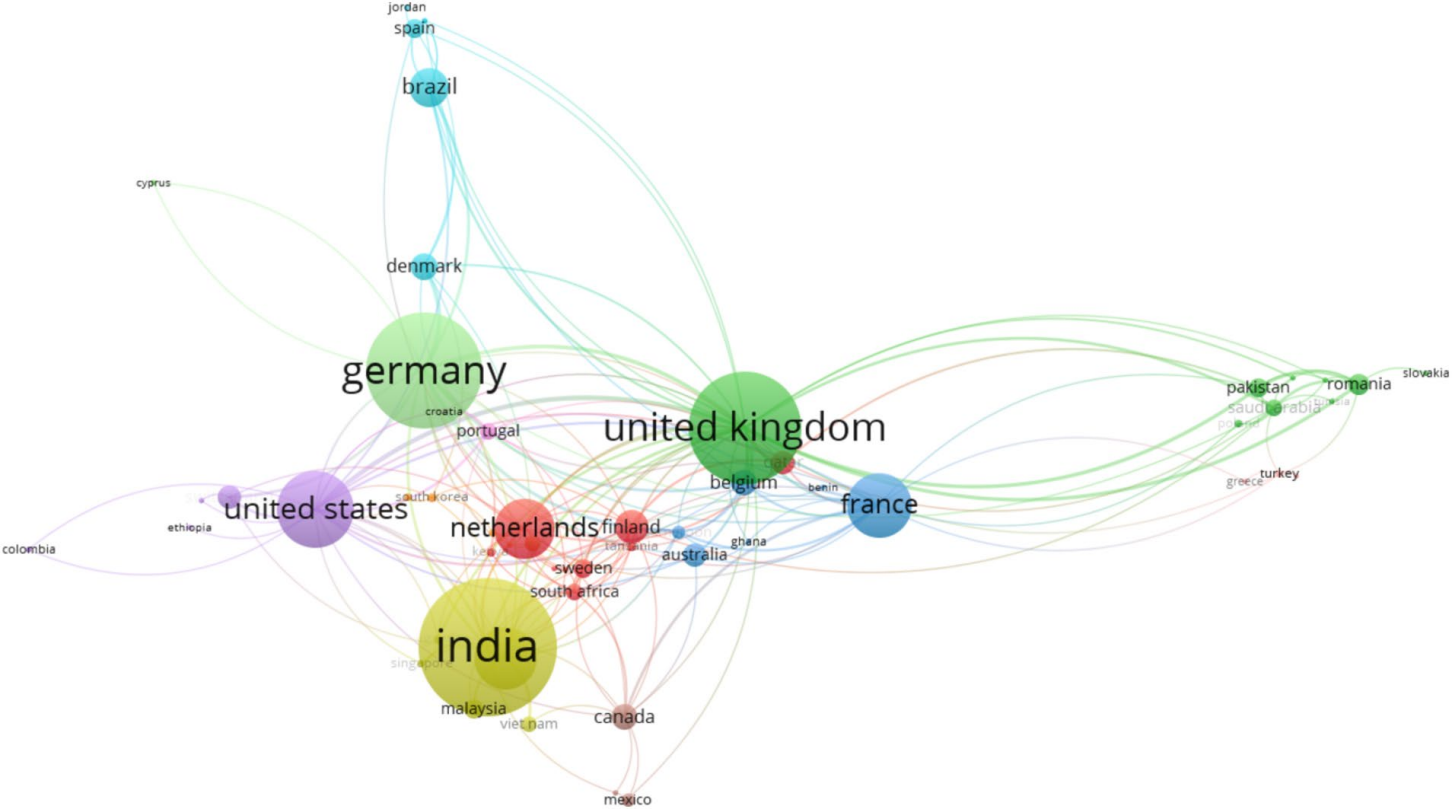
Cooperation network between countries during 2012–August 2024.

Figure 2 shows the academic collaboration among researchers from different countries from 2012 to August 2024. Each circle represents a country, and the size explains the number of articles published. Lines that are connected mean cooperation between both countries. It shows that there was close cooperation among many countries. India, United Kingdom and Germany showed the most relations, with cooperation with countries.

Despite the growth of frugal innovation, it is necessary to know in more detail what approaches are being investigated, looking at specific sectors and actors in the system. In this sense, the present study focuses on analyzing what has been researched. It proposes strategies for sustained development of frugal innovation, considering the various components such as legal, commercial, social, and economic aspects. A literature review allows for an in-depth understanding of frugal innovation, which enables researchers and scholars to understand how frugal innovation relates to other streams of thought and research approaches. That is, it helps to understand the context, provides theoretical grounding, identifies gaps in knowledge, and contributes to the knowledge of the relevance of research in the field.

## Materials and methods

### Design

The present research employs a mixed approach with a concurrent nested multi-level design. Two types of reviews are carried out to achieve the objective. First, a bibliometric review examines the evolution of publications related to frugal innovation. In addition, a desk review analyzes the evolution of the components and instruments used by the literature in the business context to measure frugal innovation.

### Search criteria

**Figure 3:**
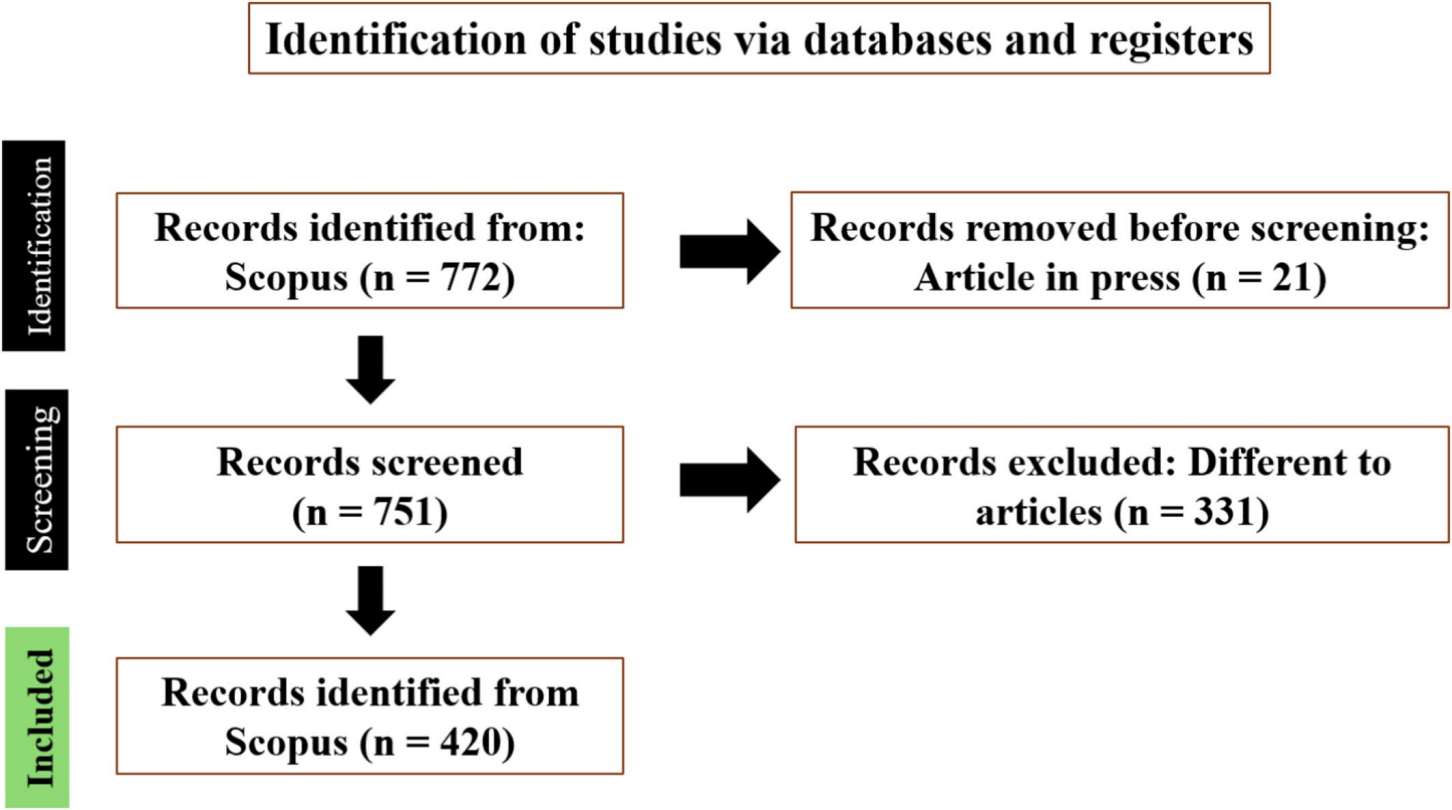
Process of article selection.

To obtain the documents corresponding to the study variable, a search was carried out on August 30th, 2024 on the Scopus website,^1^ and the algorithm was used: TITLE-ABS-KEY (“frugal innovation”) AND (EXCLUDE (PUBSTAGE, “aip”)) AND (LIMIT-TO (DOCTYPE, “ar”)) AND (EXCLUDE (PUBYEAR, 2024)). Figure 3 shows the process and steps of article selection.

Seven hundred seventy-two papers were found. Then, the publications “in press” (21) were eliminated. This way, 751 publications were evaluated, and only articles were selected. Therefore, only article-type documents were selected; thus, 420 were part of the study.

### Bibliometric analysis

The articles found were downloaded in CSV format to systematize them. Initially, the VOSviewer software was used; however, no results that showed trends were obtained because the term academic is starting to generate interest in the academic community. Consequently, frequency tables and time series graphs were used. A review was carried out to analyze the evolution and trends in the publication of scholarly articles. The journals that had published the most articles, the authors with the most publications, and the most cited articles were examined and analyzed by country and research variables.

### Simple content analysis and literature review

Articles published in journals indexed in Scopus were downloaded to review and distinguish between them. After selecting the articles, information was extracted to carry out the literature review. The definitions and components used in the selected studies were examined during this process. In addition, research that included psychometric validations of the instruments used was prioritized.

## Results

### Publications indexed in Scopus

Firstly, the number of publications indexed in Scopus on Frugal Innovation is shown. Between 2012 and 2024, 420 academic papers explicitly focusing on “frugal innovation” were identified in the Scopus database. This analysis shows that the first works related to frugal innovation date back to 2012; from then on, there is evidence of a progressive increase in academic interest in this topic, a phenomenon that becomes particularly relevant from 2020 onwards (Table 4). These findings provide a quantitative assessment of current research in this field.

**Table 4:** Articles on frugal innovation indexed in Scopus.

Year	Articles	Cites
2012	4	0
2013	8	4
2014	16	17
2015	15	39
2016	23	133
2017	22	232
2018	38	406
2019	26	463
2020	45	767
2021	53	1,226
2022	58	1,819
2023	60	2,053
2024	44	2,156

Algorithm used TITLE-ABS-KEY (“frugal innovation”) AND (EXCLUDE (PUBSTAGE, “aip”)) AND (LIMIT-TO (DOCTYPE, “ar”)).

In addition, the researcher with the largest number of articles on Frugal Innovation is Hossain, whose contribution is reflected in 15 articles focused on this field. In second place is Brem, with 11 articles related to this topic (see Table 5). Both authors have made significant contributions to research in Frugal Innovation, and their studies are widely recognized within the academic community.

**Table 5:** Authors with the highest number of articles.

N°	Authors	Articles	H-Index	Institution
1	Hossain, M.	15	23	Qatar University, Qatar
2	Brem, A.	11	43	Universität Stuttgart, Germany
3	Agarwal, N.	8	15	Universität Stuttgart, Germany
4	Le, P.B.	7	27	Hanoi University of Industry, Vietnam
5	Le Bas, C.	7	16	Institute of Sustainable Business and Organization, France

Furthermore, it is essential to note that India has the highest volume of articles, with 70 publications, followed by India and the United Kingdom, with 42 and 39 registered contributions, respectively. These data denote the significant attention that has been directed to this area in the countries and underline its importance in the research agenda (Table 6).

**Table 6:** Countries with the highest volume of articles.

N°	Countries	Articles
1	India	70
2	Germany	59
3	United Kingdom	57
4	United States	39
5	France	33
6	China	32
7	Netherlands	31
8	Brazil	20
9	Finland	17
10	Denmark	14

The institutions that have published the most articles on frugal innovation are shown in Table 7.

**Table 7:** Institutions with the highest volume of articles.

N°	Institutions	Articles
1	Friedrich-Alexander-Universität Erlangen-Nürnberg	12
2	Qatar University	11
3	Delft University of Technology	10
4	Erasmus Universiteit Rotterdam	10
5	Universität Stuttgart	9

On the other hand, the academic journals that have published the most articles on frugal innovation are shown in Table 8.

**Table 8:** Academic journals indexed in Scopus that published the most articles on frugal innovation.

No	Journals	Articles	CiteScore 2023	Editorial	H-Index	Country
1	Sustainability	23	6.8	MDPI	169	Switzerland
2	Journal of Cleaner Production	19	20.4	Elsevier	309	United Kingdom
3	International Journal of Technology Management	12	2.7	Inderscience	66	United Kingdom
4	IEEE Transactions on Engineering Management	12	10.3	IEEE	112	United States
5	Technology of Society	9	17.9	Elsevier	88	United Kingdom
6	Technological Forecasting and Social Change	9	21.3	Elsevier	179	United States
7	European Journal of Development Research	9	5.7	Springer	61	United Kingdom
8	Technovation	8	15.1	Elsevier	159	United Kingdom
9	International Journal of Innovation Management	6	3.7	World Scientific	58	Singapore
10	International Journal of Innovation Science	5	6.3	Emerald	26	United Kingdom

### Thematic analysis

Seven hundred seventy-two publications were found in the Scopus database in the period January 2012–August 2024. Leaving aside the 21 that were in press and the 331 that were not articles, 420 articles were obtained to be evaluated. The search found that the academic terms studied and frugal innovation are sustainability, sustainable development, capabilities and social entrepreneurship (see Table 9).

**Table 9:** Main variables related to frugal innovation.

Search terms	Quantity	Authors
Frugal Innovation AND Sustainability (Trad. Sustainability)	11	Molina-Maturano et al. (2020), Hossain et al. (2021), Albert (2022), Dima et al. (2022), Endres et al. (2022), Santos-Vijande et al (2022), Waqas et al. (2022), Pedroso et al. (2023), Tanguy et al. (2023), Ebolor et al. (2022), Specht et al. (2024).
Frugal Innovation AND “Sustainable performance.” (Trad: Sustainable development)	14	Dressler and Bucher (2018), Rosca et al. (2018), Arocena and Sutz (2021), Iqbal et al. (2021), Norese et al. (2021), Ebolor et al. (2022), Hossain et al. (2023), Woźniak and Wereda (2023), Dost and Umrani (2024).
Frugal Innovation AND “Capabilities” (Trad: Capabilities)	12	Reinhardt et al. (2018), Wang et al. (2018), Adhikari and Momaya (2021), Borchardt et al. (2021), Shivdas et al. (2021), Chatterjee et al. (2022), Santos et al. (2022), Jiraphanumes et al. (2023), Rossetto et al. (2023), Chakravarty and Gómez (2024).
Frugal Innovation AND “Artificial intelligence” (Trad: Artificial intelligence)	7	Subashini et al. (2019), Tiwari (2021), Ebolor et al. (2022), Kronemeyer et al. (2023), Saleem et al. (2023), Qin (2024), Molfino et al. (2024), Govindan (2024), Saleem et al. (2024).
Frugal Innovation AND “Social Entrepreneurship” (Trad: Social Entrepreneurship)	8	Igwe et al. (2020), Mishra (2021), Goyal (2021), Ananthram et al. (2024), Hossain (2022), Nilsson et al. (2022), Shahid et al. (2023), Tselepis and Nieuwenhuizen (2023), Berndt et al. (2024).

### Frugal innovation and sustainability

The research investigated different aspects of the connection between frugal innovation and sustainability, from impact assessment to application in specific contexts, such as agriculture, water, and the development of sustainable cities. These studies explore how frugal innovation practices can help reduce environmental impact and promote more efficient resource use. Sustainability in frugal innovation can include adopting cleaner technologies, reducing waste, conserving natural resources, and promoting socially responsible business practices.

Frugal innovation can promote sustainability and address global challenges to generate economic growth by helping to meet social needs and protecting the environment focused on the Sustainable Development Goals (Albert, 2022). There is a growing interest in analyzing business sustainability and consumer behavior within the context of frugal innovation, where importance is given to sustainable development measures and performance constructs (Dima et al., 2022). Within alternative scenarios for future cities that are integrated into intelligent infrastructure and governance, a retrospective approach is given for future urban planning through frugal innovation implementing cleaner production methods (Ebolor et al., 2022). Due to climate change, there has been a shift in the way innovations are evaluated. Now, the focus is on their sustainability. The term frugal business has a more significant impact in emerging markets that focus on the unmet market needs of lower-income people; Hossain et al. (2021) identify three types of constraints (resources, institutional and expansion) and demonstrate that affordable products can be provided for low-income clients, generating profits for inclusive growth.

In 2020, the COVID-19 pandemic had a global impact, and frugal innovation aided resource-scarce environments. Using two critical capabilities focused on market learning and organizational ambidexterity was essential to improve the innovation capacity of companies in low-resource markets (Santos-Vijande et al., 2022). The economic, social and environmental benefits of frugal innovations have attracted the attention of companies, government institutions and researchers. However, there are geographical areas with limited data; in the research of Molina-Maturano et al. (2020), the results showed that frugal innovations are related to the concepts of catalytic and social innovation concerning Sustainable Development Goals. They showed that neither case violated any of the 17 goals; they had a neutral impact on 33 and 48% of the SDGs and positively impacted everyone.

In South America, specifically in Brazil, the concept of frugal innovation has recently been worked on and is characterized as a new path for developing products under different limitations in many communities. The frugal innovation play a relevant role in idea creation, development and product marketing (Pedroso et al., 2023). Opposing techno-optimism, low-tech solutions have gained popularity in recent years for addressing environmental crises.

The low-tech movement is not just about switching to sturdy and less power-consuming gadgets. Although resource efficiency and material reuse are crucial components of low-tech practices, the most commonly emphasized principle in the literature and among practitioners is technical appropriation (Tanguy et al., 2023). Climate change and poverty are major global issues. The United Nations introduced the SDGs to encourage socially responsible business practices. However, implementing sustainability practices is more challenging for small and medium-sized enterprises (SMEs), resulting in compromised financial, social and environmental performance.

### Frugal innovation and sustainable development

The studies carried out with these variables focus on various aspects, from the product development process to the contribution to the SDGs and inclusion in development research. Five empirical studies and nine literature reviews were found. Assessing sustainability-oriented to SDGs is a complex but valuable approach. To manage the complexity of the SDGs, Dressler and Bucher (2018) showed an interrelationship between frugality and sustainability of an innovation. The main challenges arise from the decrease in sustainability and the increase in inequality, and their impacts depend mainly on how the tension between economic growth and environmental protection can be managed; if universities are the main actors, they could improve advanced knowledge to foster frugal innovation (Arocena and Sutz, 2021).

Achieving the Sustainable Development Goals (SDGs) is a race against time. Limited resources require frugal, affordable, user-friendly, ecologically sustainable, and socially inclusive solutions. Innovations in energy and alternative resources can be a game-changer, with sustainability and cost-effectiveness being key (Ebolor et al., 2022). Ensuring sustainable development is a critical issue for businesses, governments, and policymakers. The focus is particularly on rural areas to achieve Sustainable Development Goals. Frugal innovation presents a promising new approach to sustainable rural development. However, the literature does not explore its specific contributions in this context (Hossain et al., 2023). Companies are increasingly focused on creating positive social and environmental impact while maintaining a strong market position. Norese et al. (2021) have suggested an evaluation model and a multi-criteria overcoming method as ways to compare, rank, and select business models based on criteria such as the scope of socio-environmental concerns, strategic orientation, and the value of partnerships. Sustainable innovation is a crucial aspect of addressing social, economic and ecological issues. Frugal innovation is an all-inclusive approach that maximizes value for customers, shareholders, and society, while significantly reducing the use of financial and natural resources, primarily in developing countries (Rosca et al., 2018). Woźniak and Wereda (2023) demonstrated that factors and activities associated with the implementation of frugal innovation processes are crucial in ensuring the security and sustainable development of companies.

### Frugal innovation and capabilities

Research has addressed different aspects of dynamic, organizational, and innovation capabilities related to frugal innovation in diverse contexts, from sustainability to export competitiveness, through adaptability in times of crisis and micro-analysis and small businesses in emerging markets. One case study, five empirical studies, and three literature reviews were found. It focuses on identifying the capabilities necessary to carry out frugal innovation successfully. These capabilities may include the ability to adapt, creativity in problem-solving with limited resources, efficient cost management, and the ability to take advantage of emerging opportunities in dynamic markets.

India is the land of frugal innovation, striving to improve its capabilities. The Assets-Processes-Performance (APP) competitiveness framework was built and expanded, and such problems were diagnosed at the company level. The results indicate that companies of Indian origin are ahead of the rest in terms of their environmentally sustainable practices and obtain better results in terms of their export competitiveness (Adhikari and Momaya, 2021). Companies that concentrate on serving impoverished and subsistence markets are likely to develop more disruptive innovations that can help reduce the final prices to consumers. The main goal of these companies is to maintain basic capabilities.

Understanding the design thinking approach to product design and its usefulness in developing frugal innovations for BOP markets (Chatterjee et al., 2022). Cost management involves planning and controlling the expenses related to running a business. Therefore, the frugal innovation (FI) approach is a noteworthy strategy as it allows for cost reduction while promoting innovation without the need for substantial investments in capital, technology, and skills (Jiraphanumes et al., 2023). In order to succeed in low-end markets, companies require a specific and interdependent set of capabilities. These capabilities include internal dimensions such as a culture of cost reduction, commitment to innovation, and the ability to scale high volumes. Additionally, interface dimensions such as understanding the needs of distant customers, iteration, and developing total solutions are necessary. Finally, external dimensions such as creating access and establishing support networks at the low-end are crucial (Reinhardt et al., 2018). A scale to measure frugal innovation was built which has three main dimensions: core functionalities, significant cost reduction, and shared sustainable commitment.

Do it yourself (DIY) ability positively impacts frugal innovation development. A mediating test has confirmed that in order to develop frugal innovation in emerging markets, companies need to have the capability of DIY during times of crisis (Santos et al., 2022). Companies operating in emerging markets are quickly building frugal innovation capabilities to take advantage of the growth potential of these markets. Shivdas et al. (2021) propose that frugal innovation capability is a composite variable with four dimensions, including value for money, acceptable quality, scalability, and marketability. The results of confirmatory factor analysis validate these dimensions and provide a gateway to explore frugal innovation capabilities and their applications. The framework uses an abductive reasoning process to provide a clear and comprehensive explanation of this divergence.

### Frugal innovation and artificial intelligence

The role of AI in driving frugal innovation and its impact on various sectors and communities cannot be overstated. It has been confirmed that both IoT and AI are valid predictors of frugal innovation (Qin, 2024) so management must incorporate both capabilities to achieve frugal innovation and beat competitors in the current technological era. Artificial intelligence has the potential to identify the specific needs and problems of developing communities much faster and with increased accuracy. It can analyze demographic, health, economic and social data to pinpoint areas where innovative and cost-effective solutions are urgently needed. This approach enables frugal innovators to focus their efforts on the most pressing problems, thus maximizing the impact of their solutions. Moreover, AI can enhance the efficiency and effectiveness of frugal solutions. For instance, in the healthcare sector, machine learning algorithms can facilitate more accurate and earlier disease diagnosis, leading to more timely and less expensive treatments. Similarly, in agriculture, AI systems can optimize the use of resources like water and fertilizers, resulting in increased productivity and reduced costs for farmers. Another significant advantage of AI-enabled frugal solutions is their scalability. Since many AI technologies are software-based and can be deployed on digital platforms, these solutions can reach many people at a low marginal cost. For instance, AI-based health apps can provide specialized healthcare to remote communities that would otherwise lack access to it.

AI algorithms could optimize production, logistics, and distribution processes, which can lead to a reduction in costs and an increase in delivery speed. This is particularly relevant in situations where resources are limited and each step is critical, such as in subsistence agriculture or natural disaster management. Another area where AI and frugal innovation can come together is developing accessible technologies for people with disabilities. By creating user-friendly interfaces and intelligent assistance solutions, we can enhance the inclusion and quality of life of individuals with different abilities. For instance, by combining smart sensors with voice recognition algorithms, visually impaired individuals can interact with technological devices more naturally and efficiently (Molfino et al., 2024). AI has the potential to enhance the predictive and adaptive capabilities of frugal solutions, which can help businesses respond more quickly and effectively to unexpected changes in the market. By analyzing real-time data, AI can identify emerging trends, predict market demands, and optimize production processes, resulting in greater resilience to disruptions. Moreover, AI can aid the integration of social and environmental aspects into frugal innovation. By utilizing data analysis techniques, AI can evaluate the impact of frugal solutions on different stakeholders and the environment. This will enable more informed and equitable decision-making. Additionally, AI can help create personalized and accessible solutions that cater to the specific needs of marginalized or resource-limited communities (Govindan, 2024).

Managers and technical experts recognize the significance of frugal innovation and can use advanced text-mining techniques to identify relevant frugal patents. Artificial intelligence can analyze large patent datasets using natural language processing and machine learning algorithms, extracting valuable insights into frugal innovations. This enables companies to identify development opportunities and apply innovative strategies efficiently. Moreover, AI can enhance the frugal patent evaluation process. By employing advanced text mining methods like search string refinement and topic modeling, AI can identify patents that demonstrate frugal attributes, concentrated in specific parts of the description or distributed throughout (Kronemeyer et al., 2023). This automated analysis capability saves time and resources while improving the assessment’s accuracy and completeness.

The potential of AI in enhancing the scalability and replicability of frugal solutions cannot be overlooked. By automating processes, reducing production costs, and improving the accuracy and reliability of technologies, AI facilitates the widespread adoption of frugal innovations in low-income communities and disadvantaged environments. It is crucial to ensure that solutions designed to address the Sustainable Development Goals (SDGs) reach those who need them the most, promoting social inclusion and equity. On the other hand, frugal innovation can drive artificial intelligence’s development and ethical application. Since frugal solutions focus on simplicity, accessibility, and sustainability, they can counter the potential risks and biases associated with AI (Ebolor et al., 2022). By prioritizing community engagement, transparency, and positive social impact, frugal innovation can guide the development of AI systems that are responsible, equitable, and ethical.

A prime example of the coming together of two trends—frugal innovation and digital transformation—can be seen in the global wind sector. Through case studies and content analysis, it has been demonstrated that combining these two trends can achieve “affordable green excellence.” Artificial intelligence (AI) plays a vital role in this context by enabling the creation of more efficient and reasonably priced wind turbines that optimize their performance while reducing their environmental impact. The technological solutions arising from this synergy benefit consumers with limited resources and a broad spectrum of users, industries, and geographies (Tiwari, 2021). The key features of these digitally frugal solutions are affordability, target specificity, and efficient resource utilization. When combined, frugal innovation and artificial intelligence can significantly promote human development in rural areas by empowering women through education. With the help of innovative and AI-based e-learning platforms, women can have better access to education, removing the traditional barriers that have restrained their participation in education (Subashini et al., 2019). This will improve the life prospects and employment opportunities of individual women and contribute to the socio-economic development of rural communities.

### Frugal innovation and social entrepreneurship

This research focuses on the intersection between social entrepreneurship and frugal innovation, exploring how social entrepreneurs apply principles of efficiency and sustainability in diverse contexts, from divided urban environments to emerging economies. Searching for the full documents in academic sources for more details on these studies is recommended. Seven literature reviews and one empirical study were found. Various research highlights the relationship between frugal innovation and entrepreneurship, especially in emerging contexts. These investigations can examine how entrepreneurs use frugal innovation approaches to overcome barriers in developing markets and promote business sustainability simultaneously. Frugal innovation is often considered an effective strategy for small and medium-sized businesses (SMEs) seeking to compete in competitive markets while meeting sustainability goals. Social entrepreneurship organizations use various strategies and processes to create social and economic value through frugal innovation approaches. These approaches involve combining the creation of profitable value with addressing social problems while ensuring their sustainability. In social entrepreneurship research, the composition-based view is extended by introducing three new concepts related to composition strategies.

Frugal entrepreneurship is a new trend that has emerged from the idea of frugal innovation. Although it is a fascinating and innovative approach, it is not well-researched. In this regard, we explore the different resources that frugal entrepreneurs use and how they use them, which fall into three broad categories: human, social, and financial resources. They deeply understand their culture and context, which they integrate into their innovation process. Frugal entrepreneurs prioritize culture and context because they are part of the same society. However, patents do not help protect their innovations, which means that they rely more on informal financing than formal financing (Hossain, 2022).

The entrepreneurial ecosystem and the institutional environment influence the development of frugal innovation and informal entrepreneurship. However, there is a lack of empirical research on African business ecosystems and the factors that lead to innovations in the informal sector. To address this, a model of determinants of frugal innovation and an informal entrepreneurship ecosystem has been proposed, which includes formal and informal rules, market access, and family as important elements. These elements help to create adequate knowledge flows, networking opportunities, and capital and resource sharing, all of which contribute to the growth of frugal innovation and informal entrepreneurship (Igwe et al., 2020). In response, a social entrepreneur restructured their manufacturing process and implemented DIY techniques to produce masks urgently needed during the pandemic. The mask-making process was a frugal innovation that was necessary due to the limited resources available. The use of design thinking in frugal innovation was established through content analysis of interviews with the social entrepreneur and their team members (Mishra, 2021).

Entrepreneurs often use build-and-bridge approaches that cross both physical and digital spaces. This can be seen in spatial bricolage, which was recently introduced to develop spatial awareness (Nilsson et al., 2022). Frugal innovation-based entrepreneurship has various social outcomes, including female empowerment, improved quality of life, and affordable healthcare for low-income customers.

Understanding the good practices of female social entrepreneurs in developing countries who can enhance their social entrepreneurial efforts through innovative problem-solving is important. However, these women have shown growth and innovation in their efforts as they have dedicated themselves to solving complex problems. Therefore, frugal innovation in the context of social entrepreneurship can become increasingly relevant in a post-pandemic world (Tselepis and Nieuwenhuizen, 2023). Entrepreneurs often use a combination of physical and digital strategies to expand their businesses. This approach is known as the “build-and-bridge” approach. Recently, the concept of “spatial bricolage” has been introduced to enhance spatial awareness (Nilsson et al., 2022). Another approach to entrepreneurship is “frugal innovation,” which can lead to various social benefits, such as women’s empowerment.

### TCCM analysis

We will review the existing literature using the TCCM framework (theory development, context, characteristics, and methodology). We will identify knowledge gaps from previous research and make proposals for future research.

### Theory development

The literature on frugal innovation prominently uses theories like resource-based theory (Suherman et al., 2024), actor-network theory (Devi and Kumar, 2018), and theory of open, inclusive innovation (Gupta et al., 2016). In the current article, we note the lack of some theories that can be used to analyze frugal innovation. The theories must be used as support for future research in empirical analysis for frugal analysis; in this way, some theoretical approach can be used as open innovation because enables frugal innovation to be explored by fostering external collaboration to develop affordable solutions. By sharing knowledge and resources, companies can identify opportunities to create cost-effective and sustainable products that respond to emerging markets, optimizing costs and accelerating the innovation process with less investment. Then the Blue Ocean Strategy can be applied to frugal innovation research by identifying untapped markets and creating value by simplifying products or services, reducing costs. By focusing on essential needs and discarding superfluous features, companies can develop accessible and innovative solutions in resource-constrained markets. Innovation diffusion theory can be applied to frugal innovation by analyzing how simple, low-cost innovations are adopted in emerging markets. This allows the study of factors such as communication, timing and social context that influence the mass adoption of frugal solutions among different user groups; also, the theory of incremental innovation can be applied to frugal innovation by investigating how small improvements and continuous adjustments enable the development of more affordable and efficient solutions, optimizing limited resources. This approach helps to identify patterns of adaptation in constrained environments, facilitating low-cost, high-utility products.

### Context

Research in frugal innovation has advanced knowledge by identifying some key factors and characteristics; however, the literature base in this field is still under construction and focuses on different aspects, making it difficult to obtain a consolidated proposal. It has been possible to identify that the studies are mainly linked to five thematic areas as follows frugal innovation and sustainability (Albert, 2022; Dima et al., 2022; Ebolor et al., 2022; Hossain et al., 2021; Molina-Maturano et al., 2020; Pedroso et al., 2023; Specht et al., 2024; Tanguy et al., 2023; Waqas et al., 2022), frugal and sustainable performance (Arocena and Sutz, 2021; Dost and Umrani, 2024; Dressler and Bucher, 2018; Hossain et al., 2023; Iqbal and Piwowar-Sulej, 2023; Norese et al., 2021; Rosca et al., 2018; Woźniak and Wereda, 2023), frugal innovation and capabilities (Adhikari and Momaya, 2021; Borchardt et al., 2021; Chakravarty and Gómez, 2024; Chatterjee et al., 2022; Jiraphanumes et al., 2023; Reinhardt et al., 2018; Rossetto et al., 2023; Santos et al., 2022; Shivdas et al., 2021; Wang et al., 2018), frugal innovation and social entrepreneurship (Ananthram et al., 2024; Berndt et al., 2024; Hossain, 2022; Igwe et al., 2020; Mishra, 2021; Nilsson et al., 2022; Tselepis and Nieuwenhuizen, 2023).

### Characteristics

Studies have used partial least squares structural equation modeling (PLS-SEM) as a statistical technique of analysis, which has made it possible to determine the direct effects (e.g., the effect of frugal innovation on business model innovation) and indirect effects (e.g., business model innovation as a mediator between frugal innovation and SME internationalization) between different variables (Saleem et al., 2024; Jayabalan et al., 2024; Qin, 2024; Al-Omoush et al., 2024; Achmad and Inrawan Wiratmadja, 2024). As an example, one report includes the effect of external sources of knowledge (0.382), internal sources of knowledge (0.425), and technological turbulence (0.225) on frugal innovation in SMEs in Pakistan (Dost et al., 2019). Another study showed the effect of sustainable leadership on frugal innovation (0.239) and the effect of frugal innovation on sustainable performance (0.367) in China and India (Iqbal et al., 2021).

### Methodology

The methodology used in the current study includes correlational design (Suherman et al., 2024) case analysis (Senyo et al., 2024; Fasnacht and Proba, 2024), mix papers (Barnikol and Liefner, 2024), longitudinal (Reinhardt et al., 2024) and theoretical articles (Cooke and Knorringa, 2024; Chakravarty and Gómez, 2024). However, the other methods remain pending for new studies. Future research can use experimental studies to evaluate the modification of variables, such as the increase in frugal innovation. It is also relevant to measure frugal innovation among customers and owners.

## Discussion

This study represents a fundamental contribution to business research by conducting a comprehensive systematic review of frugal innovation, a variable with significant applications in future ventures. The research aims to close gaps in the academic literature and analyze the main variables associated with frugal innovation, including sustainability, sustainable development, capabilities and social entrepreneurship. A fundamental aspect of this research is its focus on publications indexed in Scopus, ensuring the quality and validity of the information collected. The research establishes a solid foundation by relying on sources indexed in Scopus, providing credibility and robustness to the knowledge generated. This methodological choice guarantees that the scientific community supports the results and conclusions derived from the review. Likewise, the research delves into the relationship of frugal innovation with key business variables, such as sustainability, sustainable performance, capabilities, artificial intelligence and social entrepreneurship.

The direct connection between frugal innovation and sustainability is examined in detail. The review of one case study, three empirical studies, and seven literature reviews explores how frugal innovation can reduce environmental impact and promote more efficient use of resources. Sustainability in frugal innovation translates into adopting clean technologies, reducing waste, and promoting socially responsible business practices. Next, the study analyzes the relationship of frugal innovation with sustainable performance, identifying five empirical studies and nine literature reviews. The interrelationship between frugal innovation and sustainability highlights challenges such as decreasing sustainability and increasing inequality. Frugal innovation emerges as essential to address the urgency of achieving the Sustainable Development Goals (SDGs), especially in rural areas. Furthermore, its crucial role in social, economic, and ecological progress in developing countries is recognized, as well as in maximizing value and reducing the use of resources.

Likewise, one case study, five empirical studies and three literature reviews are identified, focusing on the capabilities necessary for success in frugal innovation. India stands out as a hub of frugal innovation. It was found that Indian companies with sustainable practices are more competitive in exports, while those focused on low-resource markets develop disruptive innovations. The importance of Design Thinking and efficient cost management in frugal innovation is highlighted as crucial for success. Additionally, the connection between social entrepreneurship and frugal innovation is addressed, with seven literature reviews and one empirical study highlighting this relationship, especially in developing markets. Frugal innovation is positioned as an effective strategy for small and medium-sized businesses (SMEs) seeking to compete in competitive markets while pursuing sustainability goals.

Frugal innovation is a disruptive strategy of great relevance in the business and social environment, especially when its application in managing sustainable products and business models is considered. In the South American context, this approach can foster entrepreneurship and address specific challenges related to informality, resource scarcity, and the need for equitable opportunities. Several vital aspects emerging from this study are discussed below. *Formalization through Frugal Innovation*. Implementing frugal innovation strategies in South America can effectively promote the formalization of ventures in an environment where informality prevails due to the lack of opportunities. The training and benefits offered to new taxpayers can strengthen the system’s sustainability and contribute to long-term economic development. *Community Participation and Design Thinking*. Generating strategic alliances with local communities based on Design Thinking allows a participatory approach to product development. This approach addresses the specific needs of communities and strengthens the local economy by offering simple, affordable and sustainable solutions.

*Extended Life Cycles and Circular Economy*. Implementing extended life cycles and circular business models by entrepreneurs can be key to minimizing environmental impact. Training and introducing affordable technologies are essential to enable sustainable and attractive production practices for end consumers. *Technology as an Ally in the Post-COVID-19 Era*. The COVID-19 pandemic has highlighted the importance of technology as an ally for entrepreneurs. The incursion into digital platforms facilitates the connection with national and international markets, but to do so, it is imperative to implement secure payment systems, efficient logistics and training in digital marketing. The findings of this study offer valuable insight for academics, practitioners and decision makers. For academics, it provides a solid foundation for future research on frugal innovation and its implications for business sustainability. For professionals, it offers practical guidance on how to implement frugal innovation strategies effectively, thereby maximizing the growth and competitiveness potential of their organizations. For decision makers, it offers a clear view of the long-term benefits of adopting a frugal approach to innovation in terms of financial profitability and the positive social and environmental impact it can generate. The connection between frugal innovation and sustainability is a topic of growing interest in the academic and business community. As the world faces increasingly urgent environmental challenges, such as climate change and biodiversity loss, there is growing recognition of the need to adopt innovative and sustainable approaches to development and economic growth. Frugal innovation offers a promising approach to addressing these challenges by focusing on solutions that are economically viable, socially inclusive, and environmentally sustainable.

One of the main advantages of frugal innovation is its ability to promote efficiency in the use of resources. By finding creative ways to do more with less, organizations can reduce their dependence on scarce and expensive resources, such as energy and materials, while minimizing their environmental impact. It requires a change of mentality at both an organizational and cultural level, as well as the ability to adapt to variable contexts and conditions. Additionally, it may require significant upfront investments in research and development to identify and develop innovative solutions. However, despite these challenges, the long-term benefits of frugal innovation far outweigh the initial costs, both for companies and for society as a whole.

*Collaboration between Sectors and Elimination of Barriers*. With the increasing adoption of frugal innovation, collaboration across sectors becomes crucial. Eliminating regulatory barriers is essential to facilitate their implementation, and promoting education about these practices contributes to a social culture of buying from the entrepreneur. *Social Impact and Evaluation in NGO Projects*. Non-governmental organizations (NGOs) play a fundamental role as trainers and intermediaries. Assessing the social impact of frugal innovation projects can influence the direction of NGOs’ efforts, allowing them to pursue strategies that generate sustainable positive change in communities. *Government State Purchasing Policies*. Government policies that promote state purchases from entrepreneurs who embrace frugal innovation can further stimulate this practice, ensuring adherence to circular economy principles and generating benefits for all actors involved. *Education and Practical Collaboration*. Ultimately, this study advocates for practical collaboration between government, academia, and entrepreneurs to harness communities’ resources for social purposes. Promoting the frugal exchange of knowledge and combining efforts can lead to innovative projects contributing to South America’s sustainable development.

### Variables and items proposed

The articles reviewed established some variables that can be measured, including examples of items for questionnaires with a Likert scale (Table 10).

**Table 10:** Variables related to frugal innovation based on the article review.

Variable	Description	Items proposed
Organizational capabilities for frugal innovation	It measures organizations’ capabilities to adapt and promote innovation with limited resources.	The items proposed include: My organization can innovate using few resources. Our company can significantly reduce costs when developing new products. Our organization takes good advantage of market opportunities with low-cost innovation. We can develop functional products with limited resources.
Technologies based on artificial intelligence	It evaluates the use of AI in organizations to improve innovation processes.	My company uses AI to optimize its production processes. Artificial intelligence has significantly improved our operational efficiency. We have implemented AI to customize solutions in our product offering. AI has enabled greater scalability of our frugal solutions.
Commitment to sustainability	It reflects the degree to which organizations are committed to sustainable practices.	My organization is committed to reducing its environmental footprint. Sustainability is a key pillar in the company’s strategic decisions. We promote the efficient use of natural resources in our operations. Our innovation practices are aligned with the Sustainable Development Goals (SDGs).
Adoption of social entrepreneurship practices	It measures how companies embrace social entrepreneurship to solve local problems.	My company implements innovative solutions to address social problems. Our frugal innovations are designed to benefit marginalized communities. Social entrepreneurship is an integral part of our business strategy. We promote local economic development through our innovations.
Adaptation to the environment.	It measures the ability of organizations to adapt to changes in the environment.	Our organization responds quickly to changes in the environment. We are flexible in adjusting our strategies according to market conditions. We adapt our innovations to the changing needs of customers. Adaptation is a key factor in our innovation.
Collaborative innovation	Assessment of collaboration between different actors to drive innovation.	We regularly collaborate with other organizations to develop innovations. Teamwork with other sectors is essential for our innovations. Our company seeks external partnerships to improve product development. We encourage open innovation in collaboration with our partners.
Sustainability performance	Measures the impact of sustainable practices on the organization’s bottom line.	Our sustainability practices have improved our financial performance.
		We have significantly reduced our environmental impact through our innovations.
		Sustainable practices have enabled us to improve our market position.
		Our innovative solutions have contributed to greater social sustainability.
Scalability of frugal innovation	Measures the ability of frugal innovations to scale to different markets.	Our frugal innovations have the potential to scale to international markets.
		The reduced cost of our solutions facilitates their expansion.
		We have adapted our frugal innovations for different contexts.
		The scalability of our solutions is a key indicator of success.
Financial performance	Measures the impact of innovations on the economic performance of the company.	The implementation of frugal innovations has improved our revenues. Our innovations have reduced operating costs significantly. We have improved our profitability thanks to frugal innovation. Frugal innovations have expanded our business opportunities.

## Conclusion

One of the most significant aspects of this research is the direct relationship between frugal innovation and sustainability. By adopting innovation practices focusing on doing more with less, organizations can reduce their environmental footprint, optimize resource use, and significantly contribute to environmental preservation that has positive implications for the company’s reputation and can translate into significant savings in the long term. Furthermore, the crucial role that frugal innovation plays in promoting social entrepreneurship is highlighted. By focusing on accessible and affordable solutions, companies can address pressing social issues, such as lack of access to essential services or resource scarcity in marginalized communities, that contribute to the well-being of society as a whole and generate opportunities for inclusive economic growth.

The direct relationship between frugal innovation and sustainability is one of the most significant aspects of this research. By adopting innovative practices focused on doing more with less, organizations can reduce their environmental footprint, optimize the use of resources and significantly contribute to the preservation of the environment, which has positive implications for the company’s reputation and can translate into savings. Significant in the long term. Frugal innovation plays a crucial role in promoting social entrepreneurship, thus highlighting the importance of this approach. By focusing on accessible and affordable solutions, businesses can address pressing social issues, such as lack of access to essential services or resource scarcity in marginalized communities, contributing to the well-being of society as a whole and creating opportunities for growth. Inclusive economic.

Furthermore, the findings of this study offer valuable insight for academics, practitioners, and decision-makers. For academics, it provides a solid foundation for future research in frugal innovation and its implications for business sustainability. For professionals, it offers practical guidance on how to implement frugal innovation strategies effectively, thereby maximizing their organizations’ growth and competitiveness potential. For decision-makers, it offers a clear view of the long-term benefits of adopting a frugal approach to innovation in terms of financial profitability and the positive social and environmental impact it can generate.

## Data Availability

The original contributions presented in the study are included in the article/supplementary material, further inquiries can be directed to the corresponding author.

## Footnotes

^1^ https://www.scopus.com/
